# Preference for Artemisinin–based combination therapy among healthcare providers, Lokoja, North-Central Nigeria

**DOI:** 10.1186/s41256-018-0092-9

**Published:** 2019-01-19

**Authors:** Sylvanus C. Welle, Olufemi Ajumobi, Magbagbeola Dairo, Muhammad Balogun, Peter Adewuyi, Babatunde Adedokun, Patrick Nguku, Saheed Gidado, IkeOluwapo Ajayi

**Affiliations:** 1 Division of Health Promotion, Department of Family Health, Federal Ministry of Health, Abuja, Nigeria; 2 Nigeria Field Epidemiology and Laboratory Training Programme, Abuja, Nigeria; 3 African Field Epidemiology Network, Nigeria Country Office, Abuja, Nigeria; 4 Department of Epidemiology and Medical Statistics, University of Ibadan, Ibadan, Nigeria; 5 Liberia Field Epidemiology Training Programme, Monrovia, Liberia

**Keywords:** Artemisinin-based combination therapy, Preference for ACT, Healthcare providers

## Abstract

**Background:**

In Nigeria, Artemisinin-based Combination Therapy (ACT) is the recommended first line antimalarial medicine for uncomplicated malaria. However, health care providers still continue the use of less efficacious medicines such as Sulphadoxine-pyrimethamine and chloroquine. We therefore determined preference for ACT (PFA) and factors associated with PFA among healthcare providers (HCP) in Lokoja, North-Central Nigeria as well as assessed healthcare providers’ knowledge of malaria case management.

**Methods:**

We conducted a cross-sectional study among physicians, nurses, pharmacists, community health officers (CHOs), community health extension workers (CHEWs) and, patent and proprietary medicine vendors (PPMVs). Interviewer-administered questionnaires were administered to collect data on respondents’ characteristics, previously received malaria case management training and knowledge of malaria treatment. Knowledge scores ≥3 were categorised as good, maximum obtainable being 5.

**Results:**

Of the 404 respondents, 214 (53.0%) were males. Overall, 219 (54.2%) respondents who received malaria case management training  included PPMVs: 79 (65.8%), CHEWs: 25 (64.1%), CHOs: 5 (55.6%), nurses: 72 (48.7%), physicians: 35 (47.3%) and pharmacists: 3 (23.1%). Overall, 202 (50.0%) providers including physicians: 69 (93.2%), CHO: 8 (88.9%), CHEWs: 33 (84.6%), pharmacists: 8 (61.5%), nurses: 64 (43.2%) and PPMVs: 20 (16.5%), had good knowledge of malaria treatment guidelines. Overall, preference for ACT among healthcare providers was 39.6%. Physicians: 50 (67.6%), pharmacists: 7 (59.3%) CHOs: 5 (55.6%), CHEWS: 16 (41.0%), nurses: 56 (37.8%) and PPMV: 24 (19.8%) had PFA. Receiving malaria case management training (adjusted odds ratio [aOR]) = 2.3; CI = 1.4 – 3.7) and having good knowledge of malaria treatment (aOR = 4.0; CI = 2.4 – 6.7) were associated with PFA.

**Conclusions:**

Overall preference for ACT use was low among health care providers in this study. Preference for ACTs and proportion of health workers with good knowledge of malaria case management were even lower among PPMVs who had highest proportion of those who received malaria case management training. We recommend evaluation of current training quality, enhanced targeted training, follow-up supportive supervision of PPMVs and behavior change communication on ACT use.

## Background

In Africa, Artemisinin-based combination therapy remains the medicine of first choice for malaria treatment in most endemic countries. Artemisinin-based combination therapy (ACT) is the most effective medicine against *Plasmodium falciparum* as resistance to other antimalarial malaria medicines has been reported [1]. In 2005, Nigeria adopted ACT for malaria treatment. Despite this adoption ineffective medicines such as Sulphadoxine-pyrimethamine and chloroquine are still used to treat malaria. Children in South-South (27.0%) and North-Central (23.0%) zones are more likely to receive ACT than children in other zones [2, 3].

In Nigeria, it is common knowledge that all cadres of healthcare providers treat malaria. A preliminary survey conducted by the principal investigator in this study showed that records of medicines used in health facilities were poorly kept, trade names of medicines, rather than generic names were written in most cases, thereby making it difficult to ascertain pharmacologic constituents of such medicines. Though ACT is effective in adults and children [4], awareness about the medicine could still be low among healthcare providers [5]. Preference for ACT among healthcare providers is influenced by price and availability of the medicine [6], health facility type [7] and knowledge of recommended antimalarial medicines [8]. Nigerian policy recommends prompt parasitological confirmation by microscopy or rapid diagnostic test (RDT) in all cases of suspected malaria before treatment. In Lokoja, there are no subsidy schemes for ACTs and patients buy ACTs which are readily available in health facilities and patent medicine vendors’ shops. Cost of services are similar in private and public health facilities and are paid for out-of-pocket. The purpose of this study was to determine preference for ACT (PFA) and factors associated with PFA among healthcare providers (HCP) in Lokoja, North-Central Nigeria, as well as assessed healthcare providers’ knowledge of malaria treatment guideline (MTG).

## Methods

### Study area

Lokoja is the capital of Kogi State in North-Central Nigeria [9]. The town has a hot and humid climate with luxuriant vegetation and poor drainage system. These make the environment conducive for breeding of Anopheline mosquitoes and for endemic transmission of plasmodium parasites. In Lokoja Local Government Area, there are 10 primary healthcare centers (PHCs), one secondary health facility (State Specialist Hospital) and a tertiary health facility (Federal Medical Centre). Residents seek treatment for malaria in government-owned primary, secondary and tertiary health facilities, and in shops owned by patent and proprietary medicine vendors (PPMVs). In PHCs, malaria is treated by community health extension workers (CHEWs) and community health officers (CHOs), while physicians do same in secondary health facilities and PPMVs treat malaria in their private shops.

### Study design and population

We conducted a cross sectional survey from March to June 2014 among physicians, pharmacists, nurses, CHEWs, CHOs and PPMVs. Study participants were healthcare providers who had treated malaria for at least 1 year before commencement of the study. Eligible participants who did not consent to the study were excluded.

### Sample size calculation

Sample size of 404 was calculated using a prevalence of 38.5% [10], precision of 5% at 95% confidence interval, assuming a non-response rate of 10%.

### Sampling by probability proportional to size (PPS)

A list of physicians, pharmacists and nurses in Federal Medical Centre and State Specialist Hospital was obtained from the Chief Medical Directors. Lists of CHOs and CHEWs were obtained from the office of the Head of Primary Healthcare Department, Lokoja local government area (LGA), while the list of PPMVs was obtained from the secretary of the association of PPMVs. From a sample frame of *N* = 914, the proportion of each cadre was determined. We allocated sample sizes to the cadres using probability proportional to size and then multiplied each proportion by the sample size to ascertain the actual number of respondents per cadre (n). Thereafter, a sampling interval was calculated by dividing “N” with “n” (N/n). The first healthcare provider was selected randomly using a table of random numbers and subsequently, every n^th^ healthcare provider was selected until the sample size of 404 was completed.

**Sampling of health care providers by probability proportional to size (PPS)**

**Table:** 

**S/No.**	**Cadre**	**No. in LGA (N)**	**Proportion**	**No. allocated (n)**	**Sampling Interval, k = N/n**
1	Doctors	155	155/914 × 404 = 68	68	2
2	Pharmacists	32	32/914 × 404 = 14	14	2
3	Nurses	403	403/914 × 404 = 178	178	2
4	Community Health Officers	8	8/914 × 404 = 4	4	2
5	Community Health Extension Workers	38	38/914 × 404 = 17	17	2
6	Patent Medicine Vendors	278	278/914 × 404 = 123	123	2
	**Total**	**914**		**404**	

### Data collection

Data on respondents’ socio-demographic characteristics, knowledge of malaria diagnosis and treatment, training on malaria case management, preference for ACTs and possible factors associated with preference for ACTs were collected by trained data collectors using pre-tested semi-structured self-administered paper questionnaires. Respondents who were absent during initial visits were revisited to collect required data.

### Outcome measures

#### Assessment of knowledge of malaria diagnosis and treatment

We asked five questions to assess knowledge of malaria diagnosis and treatment among respondents. Correct response to each question was scored 1 and 0 if incorrect. Respondents who answered three or more questions correctly (≥3 points) were categorized as having good knowledge while respondents with fewer correct responses were categorized as having poor knowledge. Respondents were asked to name recommended medicines for uncomplicated malaria, malaria in pregnancy, severe malaria and intermittent preventive treatment of malaria in pregnancy. They were also asked to mention two tests for confirmation of suspected malaria cases.

#### Assessment of preference for ACT

In the questionnaire, we described scenario of a client with fever who had been diagnosed with uncomplicated malaria. The respondents were requested to write down the name, dosage and duration of medicine they would use to treat such client. Preference for ACT was defined as choice of an ACT for treatment of a case of malaria at any given time.

#### Assessment of factors associated with preference for ACT

Literature revealed factors that influenced preference for ACT. We asked questions to determine if such factors influenced healthcare providers’ preference for ACT and these factors included: cost of malaria medicines, co-existing illnesses, clients’ test results, ease of administration of antimalarials, malaria severity, clients’ weight, clients’ requests, antimalarials availability, antimalarials advertisement, antimalarials approval status and socio-demographic characteristics of respondents. These factors constituted our independent variables.

### Data analysis

Data were entered, cleaned and analyzed using Epi Info version 7 and summarized using percentages and means. The outcome variable was Preference for ACT (PFA). Chi square test was used to test relationship between categorical variables and outcome variable for bivariate analysis. Factors that were significant at bivariate analysis were further subjected to multivariate analysis (multivariate logistic regression). Results were declared significant at *p*-value < 0.05.

#### Assumptions

We assumed that a healthcare provider who was approached without prior notice to write down name, dosage and duration of medicine he/she regularly uses to treat uncomplicated malaria will give a correct response if he was in habit of treating clients with such medicine. We also assumed that respondents could volunteer such information if assured that their responses would not attract sanctions.

## Results

### Socio-demographic characteristics of respondents

The mean age of the respondents was 36.9 years (standard deviation: 9.2 years); 214 (53.0%) were males and 300 (74.4%) were married. There were more nurses, 148 (36.6%) and private medicine vendors: 121 (30.0%) than other cadres while CHOs constituted 2.2% (*n* = 9) of the respondents. Half, 207 (51.2%), had treated malaria patients for 5 years or less (Table 1).

**Table 1:** Socio-demographic characteristics of healthcare providers, Lokoja, Nigeria (*N* = 404)

Characteristics	Frequency	Percentage
Age group (years)		
15–24	21	5.2
25–34	167	41.3
35–44	126	31.2
45–54	73	18.1
≥ 55	17	4.2
Sex		
Male	214	53.0
Female	190	47.0
Marital status		
Ever Married	300	74.4
Single	104	25.6
Cadre		
Nurses	148	36.6
PMVs	121	30.0
Physicians	74	18.3
CHEWs	39	9.7
Pharmacists	13	3.2
CHOs	9	2.2
Length of work experience (years)		
1–5	207	51.2
> 5	197	48.8

### Knowledge of malaria case management among respondents

Half, 202 (50.0%) of the respondents had good knowledge of malaria case management. By cadre, 69 (93.2%) physicians, 64 (43.2%) nurses and 20 (16.5%) PPMVs had good knowledge of malaria case management. Two hundred and seventy two (67.3%) respondents knew the recommended medicine for treatment of uncomplicated malaria. Fewer than half 173 (43.3%) of the respondents knew the recommended medicine for malaria in pregnancy. Two thirds 243 (61.1%) did not know the recommended medicine for treatment of severe malaria. A quarter, 102 (25.3%) knew the tests for confirmation of suspected malaria and 309 (76.5%) respondents knew the recommended medicine for intermittent preventive treatment of malaria (Table 2).

**Table 2:** Knowledge of malaria case management among healthcare providers, Lokoja (*N* = 404)

S/No.	Knowledge Area	Correct response n (%)	Incorrect response n (%)
1	Recommended drug for treatment of uncomplicated malaria	272 (67.3)	128 (32.7)
2	Recommended drug for treatment of malaria in pregnancy	173 (43.3)	227 (56.7)
3	Recommended drug for treatment of severe malaria	157 (38.9)	243 (61.1)
4	Confirmatory tests for suspected malaria	102 (25.3)	298 (74.7)
5	Recommended drug for intermittent preventive treatment of malaria in pregnancy	309 (76.5)	91 (23.5)

### Preference for ACT and associated factors 

Overall, 160 (39.6%) healthcare providers preferred ACT. Physicians: 50 (67.6%), pharmacists: 7 (59.3%) CHOs: 5 (55.6%), CHEWS: 16 (41.0%), nurses: 56 (37.8%) and PPMV: 24 (19.8%) had PFA. We ascertained if certain factors influenced preference for ACT. These factors included cost of malaria medicines, co-existing illnesses, clients’ test results, ease of administration of antimalarials, malaria severity, clients’ weight, clients’ requests, antimalarials availability, antimalarials advertisement, antimalarials approval status and socio-demographic characteristics of respondents. These factors constituted our independent variables. Data were not collected on non-ACTs used for malaria treatment as only trade names of such medicines were documented which made it impossible to ascertain their pharmacological constituents. At bivariate analysis, Being ever married was significantly associated with preference for ACT (Unadjusted OR = 0.4, CI = 0.3 – 0.7) just as having worked for less than 5 years (Unadjusted OR = 0.4, CI = 0.3 – 0.7). Physicians were found to be 4.4 times more likely to prefer ACTs than other healthcare providers and the association was statistically significant (Unadjusted OR = 4.4, 95% CI = 2.5 – 7.5) [Table 3].

**Table 3:** Association between socio-demographic characteristics of respondents and Preference for ACT, Lokoja, North-Central Nigeria

Characteristics	No preference for ACT *N* = 244 n (%)	*Preference for ACT N* = 160 n (%)	X^2^	Unadjusted OR (95% CI)	*p*-value
Age group (years)					
< 35	118 (57.3)	88 (42.7)	1.5	0.8 (0.5 – 1.1)	0.230
≥ 35	126 (63.4)	72 (36.4)		1 (ref.)	
Sex					
Male	111 (58.4)	79 (41.6)	0.4	0.9 (0.6 – 1.3)	0.510
Female	133 (62.1)	81 (37.9)		1 (ref.)	
Marital status					
Ever Married	166 (55.3)	134 (44.7)	11.68	0.41 (0.3 – 0.7)	< 0.001
Single	78 (75.0)	26 (25.0)		1 (ref.)	
Cadre					
Non Physicians	197 (67.7)	94 (32.3)	29.2	4.4 (2.5 – 7.5)	< 0.001
Physicians	24 (32.4)	50 (67.6)		1 (ref.)	
Practice Duration(years)					
< 5	120 (52.2)	110 (47.8)	14.3	0.4 (0.3 – 0.7)	< 0.001
> =5	124 (71.3)	50 (28.7)		1 (ref.)	

Ref: reference category =1

Significant *p*-value < 0.05

As shown in Table 4, Healthcare providers whose choice of antimalarials for their patients were influenced by patients’ ability to pay for such antimalarials (Unadjusted OR 1.74, CI = 1.2–2.6) availability of antimalarials (Unadjusted OR: 2.0, CI = 1.3–3.9) and route of administration of such antimalarials (Unadjusted OR: 2.0, CI = 1.3–3.1) were more likely to prefer ACT. The associations were statistically significant. In addition, those who were trained on case management of malaria (Unadjusted OR: 1.6, CI = 1.0–2.4) and those who had good knowledge (Unadjusted OR: 5.4, CI = 3.4–8.3) were significantly more likely to prefer ACTs.

**Table 4:** Association of patient and human resource related factors with preference for ACT, Lokoja, North-Central Nigeria

Characteristics	No Preference for ACT, *N* = 244 n (%)	Preference for ACT, *N* = 160 n (%)	X^2^	Unadjusted OR (95% CI)	*p*-value
Drug treatment influenced by patients’ age	223 (59.6)	151 (40.4)	0.9	1.6 (0.7–3.5)	0.360
Drug treatment influenced by patients’ weight	214 (59.4)	146 (40.6)	0.9	1.5 (0.8 – 2.9)	0.340
Malaria severity influenced choice of antimalarial medicine	206 (58.5)	146 (41.5)	3.4	1.9 (1.0 – 3.7)	0.060
Patients' requests influenced choice of antimalarial medicine	91 (62.8)	54 (37.2)	0.4	0.9 (0.6 – 1.3)	0.530
Co-morbidity influenced choice of antimalarial medicine?	161 (57.7)	118 (42.3)	2.4	1.5 (0.9 – 2.3)	0.120
Patients' test results influenced choice of medicine	207 (58.6)	146 (41.4)	3.05	1.86 (1.0 – 3.6)	0.080
Patients’ ability to pay influenced antimalarial medicine given?	113 (54.1)	96 (45.9)	6.71	1.7 (1.2 – 2.6)	0.009*
Treatment influenced by ACT advertisement	86 (65.2)	46 (34.8)	2.6	0.7 (0.4 – 1.1)	0.110
Medicines availability influenced drugs received by patients	188 (57.1)	141 (42.9)	7.1	2.0 (1.3 – 3.9)	0.007*
Government-approval influenced choice of antimalarial medicine	227 (59.7)	153 (40.3)	0.7	1.6 (0.7 – 4.0)	0.390
Route of administration influenced choice of antimalarial medicine?	149 (55.2)	121 (44.8)	8.6	2.0 (1.3 – 3.1)	0.003*
Previous training on malaria diagnosis and treatment guidelines	122 (55.7)	97 (44.3)	1.6	1.0 – 2.4	0.039*
Good knowledge of malaria case management	84 (41.6)	118 (58.4)	5.4	3.4 – 8.3	< 0.001*

*Indicate the significant *p*-value

### Multivariate analysis of factors associated with preference for ACT among healthcare providers, Lokoja

At multivariate analysis, only two of the factors significant at bivariate analysis remained independently associated with PFA. Healthcare providers trained in malaria case management were twice more likely to prefer ACT than those who were not trained and the association was statistically significant (Adjusted OR = 2.3, 95% CI = 1.4 – 3.7). Healthcare providers who had good knowledge of malaria diagnosis and treatment were four times more likely to prefer ACT when compared with those without good knowledge (Adjusted OR = 4.0, 95% CI = 2.4 – 6.7), see Table 5.

**Table 5:** Predictors of Preference for ACT among healthcare providers, Lokoja, North-Central Nigeria

Characteristics	Adjusted OR	95% CI	*p*-value
Medicines availability influenced drugs received by patients	1.2	0.6 – 2.4 -	0.610
Patients’ ability to pay influenced drug treatment	1.5	0.9 – 2.4 -	0.120
Ever trained on guidelines**	2.3	1.4 – 3.7 -	0.008*
Healthcare provider other than doctors	0.5	0.3 – 1.0	0.052
< 5 duration of practice (years)	0.7	0.4 – 1.3	0.053
Good Healthcare providers’ knowledge of guidelines	4.0	2.4 – 6.7	< 0.001*

*Significant at *p* < 0.05

**Guidelines for diagnosis and treatment of malaria

## Discussion

This study was carried out to identify an important gap in the knowledge of case management of malaria and factors that may influence healthcare providers to prefer the recommended antimalarial medicines, ACT. The study had representation of the different cadres of healthcare providers proportionately. The findings are discussed.

### Knowledge of malaria case management among respondents

In this study, the health workers were mostly physicians which is expected as they constituted the highest proportion of healthcare providers in the state. It was only half of the respondents who had overall good knowledge of malaria case management and this was largely contributed to by the physicians as almost all of them (93.2%) demonstrated good knowledge. This was followed by nurses (43.2%) and least was PPMVs (16.5%). The domain the highest proportion of respondents demonstrated correct knowledge was knowing the name of medicine for Intermittent Preventive Treatment (IPT) of malaria in pregnancy (76.5%) followed by knowing the recommended medicine for treatment of uncomplicated malaria (67.3%) while the least was knowing the confirmatory test for malaria (25.3%). The high level of knowledge of IPT could be attributed to the fact that the drug used for IPT, Sulphadoxine-pyrimethanine is a well-known drug among healthcare providers and many of them also provide antenatal care. In addition, the PPMVs stock SP and would be aware of the various uses. The high proportion of those who knew the recommended medicine for treatment of uncomplicated malaria in this study is not surprising as all cadres of healthcare providers engage in treatment for uncomplicated in Nigeria either as healthcare provider or caregiver. On the contrary, diagnosis of malaria is usually done by the laboratory scientists or technicians who were not included in this study. However, the poor knowledge of RDT must have been contributed to by PMMVs although our data was not disaggregated by cadre. Studies have demonstrated high proportion of physicians and formal healthcare workers having correct knowledge of diagnosis [11–13] and poor knowledge among PPMVs [14] who usually refer clients to laboratory test. The use of malaria rapid diagnostic test (mRDT) recommended for informal sector including PPMVs is still not well established in Nigeria and was not promoted until recently [15]. Since the PPMVs do not have correct knowledge of diagnostics especially mRDT which is required for prescribing ACT, they are not likely to prefer ACT for treatment of malaria. This high level of ignorance is of concern because healthcare providers who do not know malaria confirmatory tests may not request for such test and could continue presumptive and unnecessary treatment of unconfirmed fevers with attendant wastes of financial resources in a low income country and increasing the risk of parasite developing resistance to antimalarial drugs.

Physicians constituted the highest proportion of healthcare providers with good knowledge of malaria diagnosis and treatment, possibly because physicians undertake mandatory continuing medical education, a requirement for renewal of their annual practicing licenses. The PPMVs are uniquely wide spread, accessible and of varied basic educational qualification [16]. Though PPMVs had largest proportion of trained respondents, they constituted the least proportion of respondents with good knowledge of malaria case management, which suggests that training alone might not be sufficient for achieving good knowledge. While the formal healthcare providers such as physicians and nurses have better opportunity of comprehensive in-service training, the informal sector though may have training, many of these are of lower quality because their lower level of education is always taking into consideration and the training focuses strictly on the services they are legally authorized to provide [14, 17]. The fact that PPMVs contributed the least to respondents with good knowledge of malaria case management also highlights a need for supportive supervision to aid knowledge retention, minimize irrational use of malaria medicines and poor treatment outcomes [14] after initial training.

### Preference for ACT and factors associated with preference for ACT

Overall, preference for ACT among healthcare providers was 39.6%. Physicians: 50 (67.6%), pharmacists: 7 (59.3%) CHOs: 5 (55.6%), CHEWS: 16 (41.0%), nurses: 56 (37.8%) and PPMV: 24 (19.8%) had PFA. It is worrisome that twelve (12) years after Nigerian government approved ACT for treatment of uncomplicated malaria, overall preference for ACT was below average, a situation which can be improved by implementation of subsidy schemes [18, 19] to lower prices and encourage preference for ACTs as cost and ability to pay are a key considerations in the choice of antimalarial drugs to prescribed as demonstrated in this study [20]. The ACTs are more expensive than the older antimalarial drugs.

Pharmaceutical companies advertise malaria medicines during clinical meetings of physicians. Though advertising medicines is associated with preference for such medicines [21], we did not find such association probably because more than eighty (80) percent of our respondents were non-physicians, who are not frequently exposed to such advertisements.

Training improves preference for ACT and quality of malaria treatment [22, 23]. This study has shown that training of healthcare providers independently predicted preference for ACT and that training outcomes depend on the cadre trained [24]. However, training alone does not automatically result in preference for ACT. For instance, out of 65.8% PPMVs trained, only 19.8% preferred ACT which suggests influence of barriers to behavior change and profit motive might have influenced preference for ACTs among PPMVs [8].

A limitation of this study was that the researchers did not review all past ‘prescriptions’ by respondents to corroborate written preferences for ACT in vignettes. However, a follow-up question requested respondents to write down names of medicines they used to treat malaria in their health facilities.

## Conclusion

Overall preference for ACT among healthcare providers was low(39.6%) while preference for ACTs and proportion of health workers with good knowledge of Malaria Case Management were even lower among PPMVs. Predictors of preference for ACT were being trained on and having good knowledge of malaria case management. However, effect of such training depended on cadre trained while having good knowledge of malaria case management alone was insufficient predictor of preference for ACT. We recommend evaluation of current training quality with emphasis on laboratory component of training, follow-up supportive supervision of PPMVs and behavior change communication on ACT use.

## Abbreviations

AFENET: African Field Epidemiology Network
CDC: United States Centers for Disease Control
CHEWs: Community Health Extension Workers
CHOs: Community Health Officers
MCMT: Malaria Case Management Training
mRDT: Malaria rapid diagnostic test (mRDT)
PFA: Preference For Artemisinin-based Combination Therapy
PHCs: Primary Healthcare Centers
PPMVs: Patent and Proprietary Medicine Vendors
